# Mixture Containing 5% Polysaccharide Extract of *Cerioporus squamosus* (Huds.) Quélet, 5% Dexpanthenol, and 0.2% Hyaluronic Acid Shows In Vitro and In Vivo Wound Healing Properties

**DOI:** 10.3390/ph18030416

**Published:** 2025-03-15

**Authors:** Jovana D. Petrović, Tamara A. Carević Milićević, Jasmina M. Glamočlija, Jelena B. Kulaš, Ivana I. Mirkov

**Affiliations:** Institute for Biological Research “Siniša Stanković”, National Institute of the Republic of Serbia, University of Belgrade, Bulevar Despota Stefana 142, 11108 Belgrade, Serbia; tamara.carevic@ibiss.bg.ac.rs (T.A.C.M.); jasna@ibiss.bg.ac.rs (J.M.G.); jkulas@ibiss.bg.ac.rs (J.B.K.); mirkovi@ibiss.bg.ac.rs (I.I.M.)

**Keywords:** *Cerioporus squamosus*, dexpanthenol, hyaluronic acid, antimicrobial activity, cytotoxicity, in vitro and in vivo wound healing properties

## Abstract

**Background:** This study explores wound healing and the antimicrobial potential of a natural formulation containing a polysaccharide extract from *Cerioporus squamosus*, hyaluronic acid, and dexpanthenol. **Methods:** Wound healing effects were assessed using HaCaT keratinocytes, while antimicrobial activity was evaluated against human skin pathogens using a microdilution assay. In vitro cytotoxicity tests ensured formulation safety, whereas in vivo wound healing was further investigated using an animal model. Gene expression analysis was performed to assess the molecular mechanisms involved. **Results:** The unique glucan composition of *C. squamosus* (15.38% α-glucans and 7.91% β-glucans) deviated from typical mushroom polysaccharide profiles, warranting further exploration of its bioactivity. In vitro mushroom polysaccharides promoted 25.35% wound closure after 24 hours, while the three-component formulation achieved 35.81% closure. Antibacterial activity showed a minimum inhibitory concentration (MIC) of 0.44–1.75 mg/mL and minimum bactericidal concentration (MBCs) of 0.88–3.50 mg/mL, while antifungal activity ranged from 0.22 to 0.44 mg/mL (MICs) and 0.44 to 0.88 mg/mL (minimum fungicidal concentration—MFC). In vivo data showed that 60% of treated wounds fully closed by day 11, despite no statistically significant difference from the control. However, gene expression analysis highlighted VEGF and collagen upregulation, indicating an enhancement of wound healing on a molecular level. **Conclusions:** The novel three-component formulation demonstrated consistent wound healing and antimicrobial properties, supporting its potential as a safe and effective treatment for chronic and acute wounds.

## 1. Introduction

A wound is damage to the skin caused by blunt trauma or an object that disrupts its barrier and impairs its functions. This injury triggers a cascade of cellular and molecular activities aimed at restoring skin integrity [1]. The wound healing process consists of several stages, including hemostasis, inflammation, proliferation (which encompasses angiogenesis, collagen deposition, granulation tissue formation, re-epithelialization, and wound contraction), and, finally, maturation and remodeling of the newly formed tissue, making it flexible and resilient to external factors [2].

Considering that the process of wound care, from the initial triage to complete healing, may involve multiple visits to healthcare professionals, and given the high incidence of daily wounds due to occupational activities, the cost of wound care can become a significant burden on any healthcare system [3]. Therefore, there is a strong need for new and efficient treatments that accelerate wound healing and allow patients to quickly return to their routine activities.

Natural products, including plants and mushrooms, have been used in wound care since ancient times due to their ability to enhance various healing aspects. Thus, given the presence of several groups of compounds with antimicrobial properties, they reduce the incidence of microbial contamination [4]. Furthermore, they exert anti-inflammatory effects, which reduce the time needed for wound closure and minimize damage to the tissue surrounding the wound. The antioxidant potential of naturally sourced substances is also quite significant, because of their ability to neutralize oxidative stress parameters accompanying wounds. Many natural matrices have been demonstrated to stimulate cell migration, thus participating in tissue regeneration [4].

Mushrooms are known for their versatile ability to promote wound healing. They have also been extensively explored for their potential applications in the cosmetic industry due to potent anti-inflammatory and antioxidant qualities, as well as their ability to address cosmetic issues like wrinkles and lines and uneven tone and texture [5]. This may be attributed to the presence of various bioactive compounds that engage several mechanisms in the process of healing and skin repair, including polysaccharides, phenolics, and lectins [6]. Among these, polysaccharide extracts (PEs) have emerged as safe and effective natural agents with significant potential. Studies have shown that PEs from *Agaricus blazei*, *Phellinus gilvus*, and *Ganoderma* spp. exhibit in vivo wound healing effects by reducing inflammation and increasing cell migration as well as mediator production [6]. Regarding *C. squamosus*, previous research has demonstrated that its methanolic extract stimulates cell migration in vitro [7]. However, while there are no data on the in vivo wound healing properties of *C. squamosus*, prior research on other species has shown consistent in vivo skin restoring effects [8]. Regardless, expanding these findings to *C. squamosus* without scientific evidence remains speculative. Thus, further experimental validation has been performed to establish its clinical relevance and therapeutic potential.

Dexpanthenol (DEX) is generally recognized as safe for skin application and is widely used for wound healing and cosmetic purposes [9]. It has good absorbing properties when administered topically, so it can readily regenerate skin and contribute to the restoration of its barrier. Furthermore, DEX shows anti-inflammatory properties both in vitro and in vivo [10].

Hyaluronic acid (HA) and its derivatives are highly valued as cosmetic ingredients because of their moisturizing and anti-aging properties. HA offers several advantages over other skincare ingredients, primarily owing to its exceptional ability to bind large quantities of water. In wound repair, HA is commonly used due to its ability to promote epidermal cell migration, whereas in cosmetic products, it is applied to fill fine wrinkles, typically at concentrations ranging from 0.2% to 1% [11].

In this study, we formulated a combination of dexpanthenol (5%), hyaluronic acid (0.2%), and mushroom-derived PE (5%) based on a review of regulatory safety assessments and the scientific literature. Dexpanthenol is known for its anti-inflammatory properties and has been shown to reduce UV-induced skin inflammation at a 5% concentration [12]. Hyaluronic acid, which is widely used in skincare, promotes hydration and wound healing, with 0.2% being a safe and effective concentration [13,14]. Mushroom-derived glucans, a key component of PE, have demonstrated skin-repairing effects, with studies supporting their use at concentrations up to 5% [15], while CIR has not established a specific concentration limit [16].

Having in mind that chronic wounds are often susceptible to microbial infections, we also evaluated the antimicrobial properties of both individual components and the formulated mixture. Bacterial biofilms and persistent infections frequently delay the healing process, highlighting the need for effective antimicrobial agents. The most commonly identified bacterial species in infected wounds include *S. aureus*, *Pseudomonas aeruginosa*, *P. mirabilis*, and *Escherichia coli* [17]. Among fungal pathogens, yeasts such as *Candida albicans*, *C. tropicalis*, *C. parapsilosis*, *C. guilliermondii*, and *C. ciferrii* are often overlooked, but play a critical role in chronic wound infections [18].

The selection of *Proteus vulgaris*, *Staphylococcus lugdunensis*, *S. epidermidis*, *Candida krusei*, *C. kefyr*, and *C. albicans* as model organisms for testing our mixture is based on their clinical relevance in wound infections and their proven ability to impede the healing process. Our choice of specific bacterial species is supported by prior findings: *S. epidermidis* has been identified in wounds [19], as well as *Proteus* spp. [20]. Furthermore, *S. lugdunensis* has emerged as a significant human pathogen capable of causing severe wound infections and abscesses, requiring careful consideration rather than being dismissed as a contaminant [21]. By selecting these bacterial and fungal species, we aim to evaluate the antimicrobial potential of our mixture against clinically relevant wound pathogens, ensuring its efficacy in preventing and managing wound infections.

Given the increasing consumer demand for natural medicine and cosmetics, the wound healing market is expected to continue growing as well, creating opportunities for research innovation and the development of novel products. Health and wellness trends, environmental awareness, and an increase in the number of patients who are sensitive to topical products are just some of the factors driving this process. Hence, developing topical formulations that contain natural bioactive compounds with proven regenerative and antimicrobial properties is crucial for improving wound care outcomes. Based on previously published research, this study explores the wound healing potential of a formulation containing 5% *C. sqamosus* PE, 5% dexpanthenol, and 0.2% hyaluronic acid. Given their complementary roles in various aspects that contribute to the adequate healing of wounds, we hypothesize that this combination will not only accelerate tissue regeneration but also reduce microbial contamination. This dual approach aims to improve wound healing efficiency while reducing the risk of infection, making the formulation a promising candidate for advanced wound care applications.

## 2. Results

The results of total, α-, and β-glucan content in the PE of *C. squamosus* showed that the *C. squamosus* sample had 23.28% total glucans, with α-glucans being present in a higher amount than β-glucans: 15.38% and 7.91%, respectively.

The results of the antimicrobial activity are presented in Table 1, and they suggest that all the individual components, as well as the mixture, showed antimicrobial properties against selected pathogenic microorganisms isolated from the skin. Among individually examined compounds, HA demonstrated the most promising antimicrobial potential, except for *S. lungdunensis* (MIC and MBC > 3.50 mg/mL). DEX showed both antibacterial and anticandidal activity against all the tested skin isolates, with *Candida kefyr*, *C. krusei*, and *C. albicans* being more susceptible to its activity than the tested bacteria. Our results also demonstrated that the PE of *C. squamosus* has antibacterial potential, with MIC values ranging from 1.75 to 3.50 mg/mL and MBC values from 3.50 to 7.00 mg/mL. Additionally, it showed promising effects against yeasts isolated from the skin, with an MIC of 0.44 mg/mL and MFC of 0.88 mg/mL.

A cytotoxicity test revealed that DEX, HA, and the PE of *C. squamosus* did not exhibit cytotoxic effects toward the HaCaT skin cell line, with IC_50_ > 400 µg/mL (Table 2).

The results of the in vitro wound healing assay are presented in Table 3. Before testing the mixture, we evaluated the wound healing properties of the individual components. The results showed that *C. squamosus* PE had beneficial wound healing effects, with 25.35% wound closure after 24 h, compared to 16.86% in the control group. As for the HA, this sample slightly delayed wound closure compared to the control: after 24 h, wound closure was 8.51% with HA, versus 16.86% without treatment. Conversely, DEX showed promising effects, with 31.26% wound closure compared to the control within the same timeframe (Table 3).

**Figure 1:**
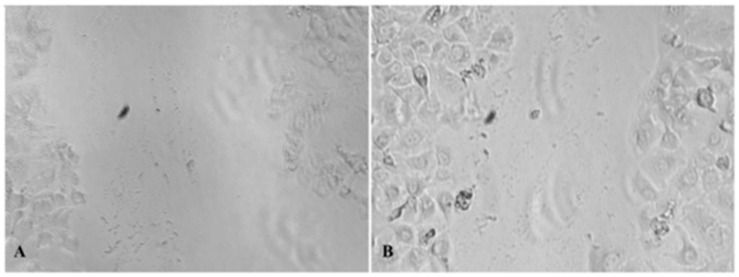
(**A**): In vitro wound healing of the mixture—0 h; (**B**): in vitro wound healing of the mixture—24 h, magnification 10× (photograph taken with Nikon camera, under Zoom 33%).

Our results indicate that the combination of three components in the proposed mixture led to a slight increase in wound closure compared to the individual components at tested concentrations, indicating possible synergistic effects (Figure 1). However, we did not investigate the underlying mechanisms of this process, as such studies are highly complex. We employed statistical analysis to evaluate whether discrepancies are/are not statistically significant, and these data are presented in Table 3. HA alone demonstrated a negative effect on wound closure compared to the control. Contrary to this, DEX exhibited a significantly higher effect, supporting its well-documented role in accelerating re-epithelialization and reducing inflammation. While showing an improvement in wound closure, PE did not reach statistical significance. Given that the effect of DEX alone was 31% and that of the mixture ~36%, we could suggest that the net effect is primarily driven by DEX, with PE providing additional complementary benefits. Despite its activity in the in vitro test, HA remained in the mixture, which will be further elaborated in the text.

**Figure 2:**
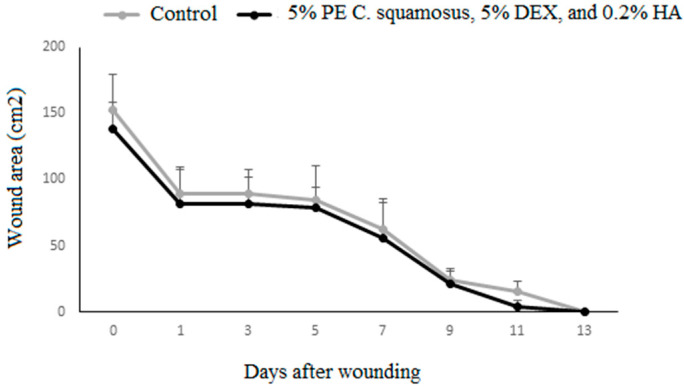
Wound closure dynamics following application of the mixture containing the PE of C. *squamosus*, DEX, and HA. Results are presented as a percentage of the initial wound areas (mean ± S.D. for a total of 9 animals per time point, per group, from two independent experiments). Data are analyzed using ANOVA with Tukey’s multiple comparisons test.

Results of the in vivo wound healing effect of the mixture on IL-1β, IL-6, and TNF expression in granulation tissue during the inflammatory phase of wound healing are presented in Figure 2. Though there is no statistically significant difference in the wound area, it should be noted that in the treated group by day 11 post-wounding, 60% of animals had completely closed wounds (in contrast to 0% in controls) (Figure 2). While this finding does not reach the threshold for statistical significance, it may still suggest a biologically relevant trend that needs further investigation with a larger sample size.

**Figure 3:**
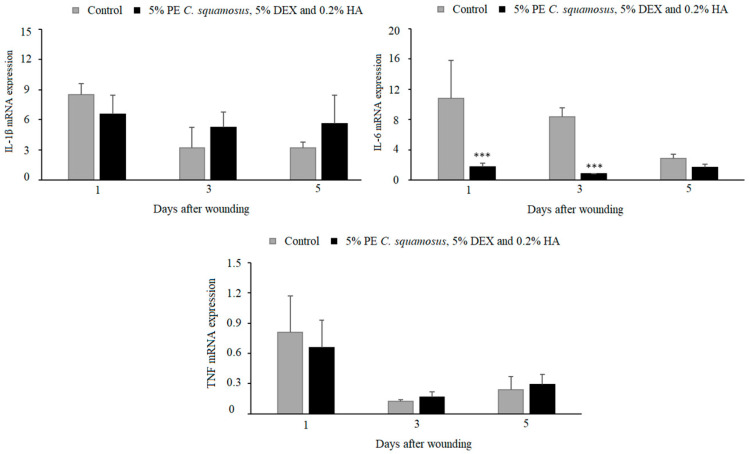
Effect of the mixture on IL-1β, IL-6, and TNF expression in granulation tissue during the inflammatory phase of wound healing. Results are presented as mean ± S.D. for a total of 9 animals per time point, per group, from two independent experiments and analyzed using ANOVA with Tukey’s multiple comparisons test. Statistically significant at *** *p* < 0.001 vs. controls.

The results we obtained for in vivo wound healing indicated no differences in IL-1β and TNF response between the control and treated group, while IL-6 was lower at days 1 and 3 post-wounding in wounds treated with the mixture containing 5% PE of *C. squamosus*, 0.2% HA, and 5% DEX (Figure 3). The effects of these components on the inflammatory phase of wound healing were assessed by measuring the expression of IL-1β, IL-6, and TNF at days 1, 3, and 5 post-wounding. Lower IL-6 in wounds treated with the mixture might contribute to the discrepancy between in vitro and in vivo results, reducing the positive effect noted in keratinocytes. It might be assumed that the effect of the mixture on other cell types present at the wound site (immune and non-immune) is absent or suppressed.

**Figure 4:**
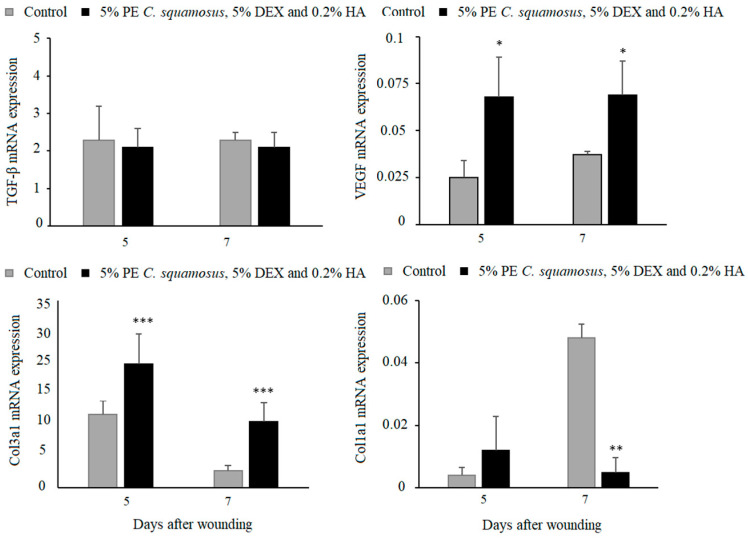
Effect of the mixture on TGF-β, VEGF, collagen 1, and collagen 3 expression in granulation tissue. Results are presented as mean ± S.D. for a total of 9 animals per time point, per group, from two independent experiments and analyzed using ANOVA with Tukey’s multiple comparisons test. Statistically significant at * *p* < 0.05, ** *p* < 0.01 and *** *p* < 0.001 vs. controls.

As for the gene expression analysis for TGF-β, VEGF, collagen 1, and collagen 3, key molecules in the proliferative phase of wound healing, results revealed similar TGF-β in both groups, higher VEGF and collagen 3, but lower collagen 1 at day 7 post-wounding in treated animals (Figure 4). Additionally, the expression of VEGF and collagen 1 and 3 began on day 3 in the treated group, compared to day 5 in the control group.

## 3. Discussion

Research has predominantly focused on the bioactivity of β-glucans over α-glucans from mushrooms, attributing numerous biological effects to them. However, while their distinct bioactivities derive from chemical differences, this does not imply that one possesses all activity while the other lacks it, as the latest research increasingly highlights the biological relevance of α-glucans as well. Recent studies have identified α-D-glucans from *Termitomyces eurhizus*, *Agaricus blazei*, and *Tricholoma matsutake*, demonstrating their immunomodulatory and antitumor effects. Regarding *C. squamosus*, no data are available on the bioactivity of its α-glucans. However, α-glucan isolated from *Polyporus grammocephalus*, a close relative of *C. squamosus*, exhibited immunostimulatory properties [22]. This result suggests that the bioactive potential of α-glucans has been mainly overlooked and is in accordance with our results as well. Furthermore, since both types of polysaccharides are almost always present in crude extracts, the possibility of synergistic interactions between these two cannot be ruled out. This aspect, however, requires further investigation. The glucan profile of the investigated mushroom is quite unusual, as previous studies have shown that mushrooms generally contain higher amounts of β-glucans compared to α-glucans, typically not exceeding 10 g per 100 g of dry weight. Thus, as reported previously, the content of α-glucans in mushrooms collected from the wild ranged from 0.70 g/100 g d.w. in *Suillus grevillei* to 8.57 g/100 g d.w. in *Russulla heterophylla* [23]. As for the β-glucan content, it ranged from 10.50 g/100 g d.w. in *Macrolepiota procera* to 34.97 g/100 g d.w. in *Tricholoma portentosum*. Commercially cultivated mushrooms showed a higher content of β-glucans as well: the lowest content of β-glucans was found in *Agaricus bisporus*—11.36 g/100 g d.w., whereas the highest content was identified in *Pleurotus ostreatus*—40.34 g/100 g d.w. Regarding glucan content in the *C. squamosus* sample, the mushroom (syn. *Polyporus squamosus*) contained 38.4 mg/g d.w. of β-glucans, though the results of α-glucan content were not presented [24]. Along with glucans, several other bioactive compounds from mushrooms contribute to wound healing as well, including polyphenols, terpenoids, proteins, and lectins. Thus, hispolon, a polyphenolic pigment from medicinal mushrooms, showed wound healing potential in diabetic conditions using in vitro and in vivo models. Hispolon promoted fibroblast migration, reduced oxidative stress, modulated inflammatory responses, and enhanced collagen synthesis, leading to accelerated wound healing [25].

The results of antimicrobial activity are in accordance with previously published data, especially regarding the antimicrobial potential of HA, as they suggest that HA of different molecular weights shows a bacteriostatic effect towards *E. coli* (ATCC 25922), *Porphyromonas gengivalis* (ATCC 33277), *Prevotella oris* (ATCC 33573), *Actinobacillus actinomycetemcomitans*, and *Proteus mirabilis* (ATCC 35508) [26]. The literature data on the antimicrobial potential of DEX indicate that it can significantly enhance the antibacterial effects of topically applied antibiotics, although no studies specifically address the antimicrobial potential of DEX per se [27]. As for the antimicrobial potential of *C. squamosus* PE, the promising results we obtained for *C. krusei* are quite important, given that this yeast causes life-threatening infection in individuals with compromised immunity [28]. Moreover, the results obtained herein are in accordance with our previously published data on the antimicrobial potential of *C. squamosus* methanolic extract, which exhibited bactericidal effects towards *E. coli* and *L. monocytogenes* (MIC in range of 0.40 to 1.50 mg/mL and MBC in range of 0.75 and 3.00 mg/mL) [29]. The combination of these ingredients did not show significant deviations from the values obtained for the individually tested components, indicating the absence of synergistic interactions, at least in terms of this type of activity. Overall, our results regarding the antimicrobial activity of individual components, as well as the mixture, are in accordance with previously published data on mushrooms, confirming their well-documented bioactive potential [30,31]. The observed inhibitory concentrations are either within the same range or even better than those previously reported for other medicinal mushrooms, demonstrating consistency with the results we obtained in our study. In certain cases, our results indicate even better antimicrobial potential, which supports the relevance of mushrooms as valuable natural sources of antimicrobial compounds. Results demonstrating the absence of toxicity of the tested components on non-cancerous cell lines are in accordance with previously published data. Thus, *P. squamosus* methanolic extracts of different origins showed no toxicity toward liver primary cell culture PLP2 at the highest concentration tested [29]. A similar effect of *P. squamosus* ethanolic extract was observed regarding activity towards HT-29 and Caco-2 cell lines [24]. Published data on cytotoxic effects of HA are somewhat in conflict. While certain data suggest an increase in cell proliferation of a mixture containing HA and acellular stromal vascular fraction [32], other data indicate a decrease in the viability of bone grafting containing osteoblastic cells in the presence of HA [33]. As for the DEX, a study demonstrated that applying 5% dexpanthenol prior to nasal decongestants can reduce the toxic effects of xylometazoline on cell growth [34]. The results we obtained herein, which suggest the safe application of the tested ingredients, are quite significant, as these components are intended for topical application on the skin [35]. With respect to the results we obtained using the in vitro wound healing model, the mixture containing 5% PE, 5% DEX, and 0.2% HA demonstrated slightly better wound healing potential than individually tested components, suggesting a potential synergistic or additive interaction, which is a known phenomenon in the bioactive properties of natural products [36]. Although the precise mechanism of action underlying this synergistic effect remains to be fully elucidated, the existing literature suggests that (i) DEX promotes the migration of keratinocytes and proliferation of fibroblasts while reducing oxidative stress and inflammation [37]; (ii) HA enhances cell migration and hydration, contributing to extracellular matrix remodeling [38]; and (iii) glucans (from PE) modulate immune responses, stimulate macrophage activity, and support collagen synthesis [39,40]. Moreover, even though previously published data suggest that the methanolic extract of this mushroom has wound healing properties in fibroblast cells (BJ-1), we obtained the first results regarding the wound healing properties of *C. squamosus* PE in HaCaT cells [7]. In general, mushrooms are known to positively influence various aspects of wound healing by reducing oxidative damage, as well as other parameters. Thus, a cream containing 3% water-insoluble (1→3)-β-glucan from *Saccharomyces cerevisiae* demonstrated wound healing properties after 90 days of application, as confirmed by a clinical study involving patients with chronic varicose vein ulcers [41]. β-Glucans are primarily associated with these effects, not only when applied topically but also when experimental animals are administered PE orally over a period of approximately 4 weeks. For instance, STZ-induced diabetic rats demonstrated improvements in wound epithelialization, collagen production, and the migration of macrophages and fibroblasts. The beneficial effects of glucans on wound healing may be attributed to the presence of receptors for glucans on human dermal fibroblasts, suggesting a direct interaction without intermediary mediation [6]. Our results show dissimilarity compared to previously published data, as our sample contained a higher proportion of α-glucans compared to β-glucans but still showed wound healing properties. The observed difference highlights the chemical diversity of naturally sourced substances and emphasizes how external factors, along with the mushroom species, can influence this diversity. The results we obtained for HA are consistent with previously published studies, which suggest that while HA is widely used in the cosmetic industry and dermatology, its wound healing effects may vary depending on the concentration used and the type of wounds being treated. Hence, it is known that primary (surgical) wounds do benefit from topical application of HA, whereas secondary wounds (with edges that cannot be approximated) show a certain delay in closing after topical application of HA [42]. Furthermore, the low concentration we used (0.2%) may also be responsible for this, though the used concentration showed the best effects within the tested mixture. In addition, the observed in vitro wound healing upon hyaluronic acid (HA) treatment may also be attributed to its molecular weight-dependent effects. Studies suggest that while low-molecular-weight HA promotes inflammation and cell migration, high-molecular-weight HA can inhibit fibroblast proliferation and keratinocyte migration, thereby delaying wound closure [43,44]. However, in combination with dexpanthenol and PE, we obtained positive effects. Given these beneficial contributions, as well as the ability of HA to reduce bacterial adhesion and biofilm formation [45], HA remained in the final formulation. As for DEX alone, our results support previously published data, which indicate that topically applied dexpanthenol supports the healing of superficial wounds [10]. Nevertheless, further investigation into specific interactions between these components and underlying mechanisms could enhance the activity of our formulation.

The results of the in vivo treatment of cutaneous wounds showed no differences in wound closure between the control and treated group. Reduced IL-6 levels in wounds treated with the mixture could help explain the difference between the in vitro and in vivo results, since this cytokine has an important role in wound healing, and the absence of IL-6 in mice was shown to impair this process [46]. This may not come as a surprise since in vitro models do not fully replicate the complexity of the tissue microenvironment present in in vivo systems [47]. While our study demonstrated improvements in wound healing parameters such as angiogenesis, collagen deposition, and inflammatory modulation, some differences between the treatment and the control group did not reach statistical significance. This may be due to factors such as sample size limitations or the complexity of wound healing, which involves multiple mechanisms. However, despite the lack of statistically significant differences in some parameters, the formulation exhibited consistent positive effects on overall healing in addition to addressing the question of microbial contamination of wounds. Overall, our obtained results suggest that the prepared mixture acts on non-immune cells through the activation of collagen production and angiogenesis, both of which contribute to re-epithelialization. The mixture containing 5% PE of *C. squamosus*, 0.2% HA, and 5% DEX is desirable in treating wounds that lack adequate epithelialization because of its ability to enhance the synthesis of new collagen in the wound [48]. Since epithelialization is a crucial aspect of wound healing, the topical application of our preparation may enhance and accelerate this process, promoting faster patient recovery [49]. This mixture may be particularly beneficial in treating chronic, slow-to-heal wounds on the lower extremities caused by varicose veins and venous insufficiency. Due to their prolonged healing time and continuous exposure to external factors, these wounds are highly susceptible to microbial contamination, which can further delay healing and require repeated medical interventions. Such wounds are prevalent among patients with underlying conditions like diabetes mellitus [50]. Also, given the growing number of patients undergoing skin rejuvenation procedures that result in wound formation as well, efficient aftercare to prevent complications and restore skin integrity is a matter of high priority. Currently available solutions, including topical antibiotics and corticosteroids, have limitations, and their use should be restricted for numerous reasons [51]. In the end, one of the most important concerns is the rise in antibiotic-resistant strains, which often result from excessive antibiotic use. Since natural products rarely cause the development of bacterial resistance, the use of the proposed mixture, which demonstrated antimicrobial properties towards skin pathogens, is particularly recommended. Given the fact that it supports inflammatory and epithelialization phases along the way, it may be considered a safe and effective topical treatment.

To summarize, many natural compounds have been recently explored for their potential in wound healing, given their promising antimicrobial, anti-inflammatory, and antioxidant properties [52]. However, studies have shown that these effects are not uniform across all stages of the healing process, and they may not simultaneously influence every phase in a positive way [53]. Thus, while certain compounds can promote collagen synthesis and angiogenesis, their impact on inflammation or re-epithelialization may vary depending on numerous factors, including the type of wound or the properties of the bioactive compounds applied. Similar trends can also be observed in other wound healing phases. As we have shown in our research, polysaccharides from mushrooms can promote collagen synthesis and VEGF factor, decrease the growth of pathogenic microorganisms isolated from the skin and in such manner as to improve skin regeneration, but have no influence of other wound healing phases at the same time. This emphasizes the necessity for targeted strategies that combine wound characteristics and the possible underlying mechanisms of natural products. This, combined with advances in biotechnology and the development of novel materials, can enhance the efficacy of natural compounds in wound healing. Thus, by integrating natural products with modern delivery systems, researchers can develop novel and efficient skin therapies and reduce dependence on commercial drugs [54].

However, some challenges remain in standardizing their use, since there is no general consensus regarding their optimal effective concentrations [53]. Our further research should prioritize the development of advanced transdermal delivery systems using this mixture to improve the effectiveness of wound care—key criteria outlined in the latest research [54,55] Additionally, in the last step, research is necessary to identify potential side effects of these natural products in clinical trials [56]. Thus, further application of natural products in wound healing requires a better understanding of their multiple roles in every stage of the wound healing process.

## 4. Materials and Methods

### 4.1. Collection of C. squamosus Fruiting Bodies

Fruiting bodies of wild growing mushroom *C. squamosus* (Huds.) Quélet (fam. Polyporaceae) were collected in Bojčinska šuma, Serbia, in 2023. They were authenticated by Jovana Petrović, PhD, Institute for Biological Research “Siniša Stanković” (IBISS), National Institute of the Republic of Serbia, University of Belgrade, Serbia. A voucher specimen has been deposited at the Fungal Collection Unit of the Mycological Laboratory at IBISS, under the collection number CS-IBRSS-2023. After collecting, fruiting bodies were cleaned of debris and sliced and frozen, after which they were lyophilized (LH Leybold, Lyovac GT2, Frankendorf, Switzerland), blended to a fine powder (20 mesh), and stored until further use.

### 4.2. Preparation of C. squamosus Polysaccharide Extract

Mushroom powder (10 g) was subjected to extraction with distilled water (200 mL) in an autoclave at 121 °C for 20 min, at 121,59 Pa, according to the modified procedure described previously [57]. After autoclaving, the suspension was cooled on ice, centrifuged at 4000× *g* for 20 min (Heraeus biofuge stratos centrifuge, Thermo electron corporation, Waltham, MA, USA), and then filtered through Whatman paper No. 4. The resulting polysaccharide extract (PE) was frozen and then lyophilized, after which the sample was stored at +4 °C.

### 4.3. Measurement of Total, α- and β-Glucan Content in PE of C. squamosus

The content of total, β-, and α-glucans in PE was measured using the enzyme kit “Mushroom and Yeast β-glucan Assay”, K-YBGL 02/2021 (Megazyme Int., Wicklow, Ireland). All the measurements were performed as described by the manufacturer’s suggestions, and all the obtained values were calculated using Megazyme Mega-Calc^TM^ software, © 2023, Neogen Corporation, available on the Megazyme website (www.megazyme.com).

#### 4.3.1. Measurement of Total Glucan Content in PE of *C. squamosus*

Briefly, total glucan content was determined using the following procedure: Mushroom PE (90 mg) was hydrolyzed with ice-cold 12 M H_2_SO_4_ (2.0 mL), after which the sample was incubated for 2 h on ice and periodically agitated (10–15 s) to ensure complete hydrolysis of the glucan. After incubation, water (10 mL) was added to the suspension in two portions with intermittent agitation. The resulting suspension was incubated in a water bath at 100 °C for 2 h, and then cooled down to room temperature. The content of each tube was quantitatively transferred using 200 mM sodium acetate buffer pH 4.5. Subsequently, 8.0 M NaOH solution (6 mL) was added to each sample; the contents were mixed, aliquoted, and then centrifuged for 5× *g* min at 13,000 rpm (Eppendorf 022620100 MiniSpin Personal Microcentrifuge, Eppendorf, Hamburg, Germany). The resulting extract was mixed with exo-1,3-β-Glucanase (100 U/mL) plus β-Glucosidase (20 U/mL) in a 1:1 ratio. The contents were mixed and then incubated at 40 °C for 60 min, after which GOPOD reagent (buffer 50 mL, pH 7.4, p-hydroxybenzoic acid and sodium azide 0.09%, 3.0 mL) was added to each tube. The tubes were once more incubated at 40 °C for 20 min, after which the absorbance of the sample was measured at 510 nm.

#### 4.3.2. Measurement of α- and β-Glucan Content in PE of *C. squamosus*

α-glucan content in PE was measured as follows: Mushroom PE (90 mg) was mixed with 1.7 M NaOH (2.0 mL) and the contents were suspended in an ice bath for 20 min. Subsequently, 1.2 M sodium acetate buffer pH 3.8 (8 mL) was added, as well as amyloglucosidase 1.630 U/mL plus invertase 500 U/mL solution in 50% (*v*/*v*) glycerol (0.2 mL). The resulting suspension was thoroughly mixed and then incubated in a water bath at 40 °C for 60 min. After incubation, the content of the tube was mixed with sodium acetate buffer (200 mM, pH 4.5 in 1:1 ratio) and then GOPOD reagent was added (3 mL). The resulting mixture was incubated at 40 °C for 20 min, after which the absorbance was measured at 510 nm. β-glucan content was calculated as the difference between the total glucan and the α-glucan content.

### 4.4. Antibacterial and Anticandidal Activity

In the present study, we used the following bacteria, *Proteus vulgaris* (B44), *Staphylococcus lugdunensis* (B43), and *S. epidermidis* (B45), as well as yeasts: *Candida kefyr* (Y289) and *C. albicans* (Y177). All the microorganisms are deposited at the Collection of the Mycological laboratory of the Institute for Biological Research “Siniša Stanković”, National Institute of the Republic of Serbia, University of Belgrade. Evaluated pathogenic microorganisms are clinical samples isolated from infected skin. The samples were collected at the Medical Military Academy (MMA), Belgrade, Serbia, from patients with skin infections, in accordance with an ethical committee of the MMA, approved under No. 4/2021. Microorganisms were identified using the Vitek MS system (bioMerieux SA, Marcy-l’Étoile, Lyon, France), which uses matrix-assisted laser desorption ionization time of flight (MALDI-TOF) to provide automated mass spectrometry microbial identification. The antibacterial and anticandidal activities of the tested samples were evaluated using the microdilution method in 96-well microtiter plates. The minimum inhibitory concentrations (MICs) and minimal bactericidal/fungicidal concentrations (MBCs/MFCs) of extracts were determined as described previously [58]. Streptomycin and ketoconazole were used as positive controls.

### 4.5. Cytotoxicity Towards HaCaT Cell Line

The cytotoxicity of the tested ingredients (PE of *C. squamosus*, DEX, and HA) towards the spontaneously immortalized HaCaT cell line was evaluated using the crystal violet test, as described previously [59]. In brief, HaCaT cells were cultured at 37 °C in an incubator with 5% CO_2_ using high-glucose Dulbecco’s Modified Eagle Medium (DMEM) supplemented with 10% fetal bovine serum (FBS), 2 mM l-glutamine, and 1% antibiotic–antimycotic (Invitrogen). Cells (1.7 × 10^4^) were seeded in a 96-well microtiter plate with an adhesive bottom. After 48 h, the cultivation medium was removed, and the cells were treated overnight with different concentrations of the tested sample. After treatment, cells were washed twice with PBS and stained for 20 min with crystal violet solution (0.4%). Subsequently, the stain was removed, and the cells were washed with tap water and air-dried. Methanol was added to dissolve the bounded residues of crystal violet, and the resulting absorbance was measured at 570 nm using a plate reader (MultiskanTM FC Microplate Photometer, Thermo Scientific, Waltham, MA, USA). Potassium dichromate (K_2_Cr_2_O_7_) was used as a positive control and PBS as a negative one. The experiment was performed in triplicate. Results are expressed as the IC_50_ (%) value in μg/mL, indicating 50% of cell viability when compared with the untreated control.

### 4.6. In Vitro Wound Healing Assay

HaCaT cells were grown to confluency, after which the cell monolayer was scraped with a sterile tip, according to Petrović et al. (2024) [60]. Floating cells were washed, and cells were incubated in reduced DMEM supplemented with 1% FBS, 2 mM l-glutamine and 1% antibiotic–antimycotic (Invitrogen), containing 250 mg/mL of the tested sample. Cell migration was monitored with a Nikon Eclipse TS2 inverted microscope (Amsterdam, The Netherlands) 24 h after the wound was initiated and treated. As a control, we evaluated wound closure under identical conditions without adding any test samples. Obtained results were presented as percentages of wound closure with the tested sample in relation to the untreated control.

### 4.7. In Vivo Experiments

#### 4.7.1. Experimental Animals Protocol

Animal treatment and experimental procedures were carried out in compliance with the EU Directive 2010/63/EU on the protection of animals used for experimental and other scientific purposes. Procedures were approved by the Veterinary Directorate, Ministry of Agriculture, Forestry and Water Management under ethical clearance number 01-52/24. To monitor the dynamics of wound closure, healthy individuals of the DA rat strain were used, which were males of eight to twelve weeks of age. Animals were housed at the IBISS in the Laboratory Animal Rearing Facility in a controlled environment (21−24 °C temperature, a 60% relative humidity, and 12 h light/dark cycle). A wound 1 cm in diameter was created on the dorsal region near the shoulders, on a previously trimmed skin surface. The subjects were divided into two groups: (i) untreated wounds and (ii) wounds treated with a mixture containing 5% PE *C. squamosus*, 5% DEX, and 0.2% HA. Wounds were treated once daily, and the dynamics of wound closure were monitored with daily measurements until healing was complete. Granulation tissue formed during wound healing was collected and homogenized in a mi-Total RNA Isolation Kit (Metabion, Martinsried, Germany) to examine the expression of factors relevant to inflammatory (IL-1β, TNF, and IL-6) and proliferative (TGF-β, VEGF, collagen 1, and collagen 3) phases of wound healing.

#### 4.7.2. Reverse Transcription and Real-Time Polymerase Chain Reaction (RT-PCR)

Total RNA (1 mg) isolated from granulation tissue using the mi-Total RNA Isolation Kit was reverse transcribed using random hexamer primers and MMLV (Moloney Murine Leukemia Virus) reverse transcriptase (Fermentas, Vilnius, Lithuania), according to the manufacturer’s instructions. Prepared cDNAs were amplified using Power SYBR^®^ Green PCR Master Mix (Applied Biosystems, Foster City, CA, USA) according to the manufacturer’s specification in a total volume of 20 µL in a Quant StudioTM 3 Real-Time PCR Instrument (96-well, 0.2 mL) (Applied Biosystems). The thermocycler conditions were as follows: a 50 °C hold step for 2 min, followed by a 95 °C step for 10 min; a 95 °C step for 15 s, followed by a 60 °C step for 60 s for 40 cycles; and melting curve phase at 95 °C for 15 s, followed by a step at 60 °C for 1 s.

PCR primers (forward/reverse) used in the study were as follows:

Β-actin: 5′−CCCTGGCTCCTAGCACCAT-3′/5′-GAGCCACCAATCCACACAGA-3′

IL-1β: 5′-CACCTCTCAAGCAGAGCA-3′/5′-GGGTTCCATGGTGAAGTCAAC-3′

IL-6: 5′-CCCTTCAGGAACAGCTATGA-3′/5′-TGTCAACAACATCAGTCCCAAG-3′

TNF: 5′-TCGAGTGACAAGCCCGTAGC-3′/5′-CTCAGCCACTCCAGCTGCTC-3′

TGF-β: 5′-CCCTGCCCCTACATTTGGA-3′/5′-ACGGTGATGCGGAAGCAC-3′

VEGF:5′-GGGCCTCTGAAACCATGAACT-3′/5′-ACGTCCATGAACTTCACCACTTC-3′;

Col1a1: 5′-ACATGCCGTGACCTCAAGAT-3′/5′-ATGTCCATTCCGAATTCCTG-3′

Col3a1: 5′-CTGGTCCTGTTGGTCCATCT-3′/5′-ATGCCATTAGAGCCACGTTC-3′

mRNA accumulation was detected in real time and the results were analyzed using Quant StudioTM Design & Analysis Software v1.4.3 (Applied Biosystems) and calculated as 2^−dCt^, where dCt is the difference between the cycle threshold (Ct) value of the specific gene and the endogenous control (β-actin).

### 4.8. Statistical Analysis

For data obtained in the CV assay, GraphPad Prism 6.01 software (Software, Inc., San Diego, CA, USA) was used, and one-way ANOVA with Dunnet’s and Tukey’s post hoc tests was applied; the level of significance was set at * *p* < 0.05. For the in vivo experiments, results were pooled from two independent experiments with four or five animals per group per experiment (a total of nine animals per group) and presented as mean ± standard deviation. Data were compared by analysis of variance (one-way ANOVA) followed by Tukey’s test (STATISTICA 7.0, StatSoft Inc., Tulsa, OK, USA), and *p*-values < 0.05 were considered significant.

## 5. Conclusions

The results we obtained in this study suggest the in vitro and in vivo wound healing potential of a novel mixture containing PE of 5% *C. squamosus*, 0.2% HA, and 5% DEX. Our glucan analysis revealed a higher proportion of α-glucans in the PE sample, highlighting the importance of both α- and β-glucans in skin repair. The tested mixture of all components showed promising wound healing results in vitro and demonstrated good antimicrobial activity against skin pathogenic microorganisms, including *C. kefyr* and *C. albicans*. Ingredients were confirmed to be safe for the skin cell line, while in vivo tests showed improvement in the production of collagen and overall epithelialization. Although the in vivo data we obtained showed promising results, they were not statistically significant when compared to the control group. Despite this, the positive trends observed, including enhanced collagen production and improved epithelialization, provide important preliminary support for the therapeutic potential of the mixture. In addition, the ability of the mixture to inhibit the growth of pathogenic microorganisms implicated in wound healing further implies that it can be beneficial in the healing of infected wounds. Further studies should include histopathological analyses, which would provide a more comprehensive understanding of the healing process. Overall, the proposed mixture not only aligns with current trends in wound management but also addresses the increasing demand for natural, sustainable, and overall effective wound care solutions. In conclusion, our study provides a strong foundation for additional exploration of this mixture’s potential in chronic wound management and encourages further research to fully assess its wound healing capacity.

## 6. Patents

The presented results are part of the patent application filed under No. P-2024/0601 in The Intellectual Property Office of the Republic of Serbia.

## Figures and Tables

**Table 1:** The antimicrobial activity of PE *C. squamosus,* HA, DEX, and the mixture containing all three components (mg/mL).

Microorganisms		PE *C. squamosus*	HA	DEX	Mixture	Streptomycin Ketoconazole
*P. vulgaris* (B44) (clinical isolate) *	MIC	3.50	0.44	1.75	1.75	0.003
	MBC	7.00	0.88	3.50	3.50	0.006
*S. lungdunensis* (B43) (clinical isolate) *	MIC	3.50	>3.50	1.75	1.75	0.003
	MBC	7.00	>3.50	3.50	3.50	0.006
*S. epidermidis* (B45) (clinical isolate) *	MIC	1.75	0.44	0.88	0.44	0.10
	MBC	3.50	0.88	1.75	0.88	0.20
*C. kefyr* (Y289) (clinical isolate) *	MIC	0.44	0.22	0.44	0.22	0.015
	MFC	0.88	0.44	0.88	0.44	0.030
*C. krusei* (Y454) (clinical isolate) *	MIC	0.44	1.75	0.44	0.44	0.015
	MFC	0.88	3.50	0.88	0.88	0.030
*C. albicans* (Y177) (clinical isolate) *	MIC	0.44	0.22	0.44	0.22	0.015
	MFC	0.88	0.44	0.88	0.44	0.030

* clinical isolates from the infected skin of volunteers, approved by the ethical committee of the MMA under No. 4/2021.

**Table 2:** Cytotoxic activity of PE *C. squamosus*, HA, and DEX (μg/mL).

Sample	IC_50_ (μg/mL)
PE *C. squamosus*	>400
HA	>400
DEX	>400
K_2_Cr_2_O_7_	83.40

**Table 3:** In vitro wound healing properties of PE *C. squamosus,* HA, DEX, and the mixture.

Tested Sample	Wound Closure After 24 h (%)	Comparison to Control	Comparison to Combination
Control	16.86 ± 3.17		
PE *C. squamosus*	25.35 ± 4.24	Not statistically significant	*
HA	8.51 ± 1.11	*	****
DEX	31.26 ± 0.97	**	*
Mixture	35.81 ± 2.14	**	

Student *t*-test, results obtained in the program GraphPad Prism9. * represents a statistically significant difference: *, *p* ≤ 0.05; **, *p* ≤ 0.01; ****, *p* ≤ 0.0001. Results are shown as mean ± SD.

## Data Availability

The data that support the findings of this study are available on request from the corresponding author, [J.P.].

## Abbreviations

The following abbreviations are used in this manuscript:

PE	polysaccharide extract
MIC	minimum inhibitory concentration
MBC	minimum bactericidal concentration
MFC	minimum fungicidal concentration
DEX	dexpanthenol
HA	hyaluronic acid
IL-1β	interleukin-1 beta
IL-6	interleukin-6
TNF	tumor necrosis factor
TGF-β	transforming growth factor-β
VEGF	vascular endothelial growth factor

## References

[1] Mapoung S., Umsumarng S., Semmarath W., Arjsri P., Thippraphan P., Yodkeeree S., Limtrakul Dejkriengkraikul P. (2021). Skin wound-healing potential of polysaccharides from medicinal mushroom *Auricularia auricula-judae* (Bull.). J. Fungi.

[2] Schultz G.S., Chin G.A., Moldawer L., Diegelmann R.F., Fitridge R., Thompson M. (2011). Principles of wound healing. Mechanisms of Vascular Disease: A Reference Book for Vascular Specialists.

[3] Harding K., Queen D. (2023). Estimating the cost of wounds for the United Kingdom and its countries. Int. Wound J..

[4] El-Sherbeni S., Negm W.A. (2023). The wound healing effect of botanicals and pure natural substances used in *in vivo* models. Inflammopharmacology.

[5] Wu Y., Choi M.H., Li J., Yang H., Shin H.J. (2016). Mushroom cosmetics: The present and future. Cosmetics.

[6] Sharifi-Rad J., Butnariu M., Ezzat S.M., Adetunji C.O., Imran M., Sobhani S.R., Tufail T., Hosseinabadi T., Ramírez-Alarcón K., Martorell M. (2020). Mushrooms-rich preparations on wound healing: From nutritional to medicinal attributes. Front. Pharmacol..

[7] Elkhateeb W., Daba G., Elnahas M., Thomas P., Emam M. (2020). Metabolic profile and skin-related bioactivities of *Cerioporus squamosus* hydromethanolic extract. Biodivers. J..

[8] Xu C., Wang F., Guan S., Wang L. (2024). β-Glucans obtained from fungus for wound healing: A review. Carbohydr Polym..

[9] Najafiasl M., Osfouri S., Azin R., Zaeri S. (2020). Fabrication, characterization and *in vivo* evaluation of dexpanthenol sustained-release nanofibers for wound healing. Polym. Test..

[10] Gorski J., Proksch E., Baron J.M., Schmid D., Zhang L. (2020). Dexpanthenol in wound healing after medical and cosmetic interventions (postprocedure wound healing). Pharmaceuticals.

[11] Juncan A.M., Moisă D.G., Santini A., Morgovan C., Rus L.L., Vonica-Țincu A.L., Loghin F. (2021). Advantages of hyaluronic acid and its combination with other bioactive ingredients in cosmeceuticals. Molecules.

[12] (2017). Cosmetic Ingredient Review. Safety Assessment of Panthenol, Pantothenic Acid, and Derivatives as Used in Cosmetics. https://www.cir-safety.org/sites/default/files/panthenol_0.pdf.

[13] Cosmetic Ingredient Review. Safety Assessment of Hyaluronates as Used in Cosmetics. 2022. https://www.cir-safety.org/sites/default/files/Hyaluronates_1.pdf.

[14] Liu J.K. (2022). Natural products in cosmetics. Nat. Prod. Bioprospect.

[15] Natakankitkul S., Homdok P., Wandee P., Krisdaphong T., Toida (2016). Development of skincare cosmetic from yeast beta-glucans. TJPS.

[16] Cosmetic Ingredient Review. Safety Assessment of Yeast-Derived Ingredients as Used in Cosmetics. 2021. https://cir-safety.org/sites/default/files/yeast092021SLR.pdf.

[17] Bessa L.J., Fazii P., Di Giulio M., Cellini L. (2015). Bacterial Isolates from Infected Wounds and Their Antibiotic Susceptibility Pattern: Some Remarks about Wound Infection. Int. Wound J..

[18] Ge Y., Wang Q. (2023). Current Research on Fungi in Chronic Wounds. Front. Mol. Biosci..

[19] Severn M.M., Horswill A.R. (2023). Staphylococcus epidermidis and its dual lifestyle in skin health and infection. Nat. Rev. Microbiol..

[20] Mordi R., Momoh M. (2009). Incidence of *Proteus* Species in Wound Infections and Their Sensitivity Pattern in the University of Benin Teaching Hospital. Afr. J. Biotechnol..

[21] Papapetropoulos N., Papapetropoulou M., Vantarakis A. (2013). Abscesses and wound infections due to *Staphylococcus lugdunensis*: Report of 16 cases. Infection.

[22] Patra S., Maity P., Chakraborty I., Sen I., Ghosh D., Rout D., Bhanja S. (2020). Structural studies of immunomodulatory (1 → 3)-, (1 → 4)- glucan from an edible mushroom *Polyporus grammocephalus*. Int. J. Biol. Macromol..

[23] Mirończuk-Chodakowska I., Witkowska A. (2020). Evaluation of Polish wild mushrooms as beta-glucan sources. Int. J. Environ. Res. Public Health.

[24] Doskocil I., Havlik J., Verlotta R., Tauchen J., Vesela L., Macakova K., Opletal L., Kokoska L., Rada V. (2016). *In Vitro* immunomodulatory activity, cytotoxicity and chemistry of some Central European polypores. Pharm. Biol..

[25] Liu Y.S., Lai M.C., Tzeng Y.C., Liu I.M. (2025). Polyphenolic Hispolon Derived from Medicinal Mushrooms of the *Inonotus* and *Phellinus* Genera Promotes Wound Healing in Hyperglycemia-Induced Impairments. Nutrients.

[26] Zamboni F., Wong C.K., Collins M.N. (2022). Hyaluronic acid association with bacterial, fungal and viral infections: Can hyaluronic acid be used as an antimicrobial polymer for biomedical and pharmaceutical applications?. Bioact. Mater..

[27] Helaly G.F., El-Aziz A.A.A., Sonbol F., El-Banna T., Lotfy N. (2011). Dexpanthenol and propolis extract in combination with local antibiotics for treatment of *Staphylococcal* and *Pseudomonal* wound infections. Arch. Clin. Microbiol..

[28] Abbas J., Bodey G.P., Hanna H.A., Mardani M., Girgawy E., Abi-Said D., Whimbey E., Hachem R., Raad I. (2000). *Candida krusei* fungemia: An escalating serious infection in immunocompromised patients. Arch. Intern. Med..

[29] Fernandes A., Petrović J., Stojković D., Barros L., Glamočlija J., Soković M., Martins A., Ferreira I.C.F.R. (2016). *Polyporus squamosus* (Huds.) Fr from different origins: Chemical characterization, screening of the bioactive properties and specific antimicrobial effects against *Pseudomonas aeruginosa*. LWT Food Sci. Technol..

[30] Alves M.J., Ferreira I.C., Dias J., Teixeira V., Martins A., Pintado M. (2012). A Review on Antimicrobial Activity of Mushroom (Basidiomycetes) Extracts and Isolated Compounds. Planta Med..

[31] Matijašević D., Pantić M., Rašković B., Pavlović V., Duvnjak D., Sknepnek A., Nikšić M. (2016). The Antibacterial Activity of *Coriolus versicolor* Methanol Extract and Its Effect on Ultrastructural Changes of *Staphylococcus aureus* and *Salmonella Enteritidis*. Front Microbiol..

[32] Mohan S.P., Palaniappan A., Nawaz M.K.K., Kripamol R., Seenuvasan R., Kumar P.R.A. (2023). *In vitro* cytotoxicity evaluation of flowable hyaluronic acid-acellular stromal vascular fraction (HA-aSVF) mixture for tissue engineering applications. J. Pharm. Bioallied Sci..

[33] Boeckel D.G., Shinkai R.S., Grossi M.L., Teixeira E.R. (2014). *In Vtro* evaluation of cytotoxicity of hyaluronic acid as an extracellular matrix on OFCOL II cells by the MTT assay. Oral Surg. Oral Med. Oral Pathol. Oral Radiol..

[34] Klöcker N., Rudolph P., Verse T. (2004). Evaluation of protective and therapeutic effects of dexpanthenol on nasal decongestants and preservatives: Results of cytotoxic studies in vitro. Am. J. Rhinol..

[35] Choi S.Y., Seop S.Y., Hyun M.Y., Yoo K.H., Kim B.J., Kim M.N., Cho J.W. (2013). Safety evaluation of topical valproate application. Toxicol. Res..

[36] Vaou N., Stavropoulou E., Voidarou C., Tsakris Z., Rozos G., Tsigalou C., Bezirtzoglou E. (2022). Interactions between Medical Plant-Derived Bioactive Compounds: Focus on Antimicrobial Combination Effects. Antibiotics.

[37] El-Seddawy F., Abdel-Maboud M., Barakat N., Hassaan M. (2023). Dexpanthenol: New Insights on Wound Healing, a Review. J. Adv. Vet. Res..

[38] Riessen R., Wight T.N., Pastore C., Henley C., Isner J.M. (1996). Distribution of hyaluronan during extracellular matrix remodeling in human restenotic arteries and balloon-injured rat carotid arteries. Circulation.

[39] Wei D., Zhang L., Williams D., Browder I. (2002). Glucan stimulates human dermal fibroblast collagen biosynthesis through nuclear factor-1 dependent mechanism. Wound Repair Regen..

[40] Minqi Q., Bing L., Dezhi G., Qi X., Yanjiao X., Qiang D., Shunqing T. (2022). Aminated β-Glucan with immunostimulating activities and collagen composite sponge for wound repair. Int. J. Biol. Macromol..

[41] Majtan J., Jesenak M. (2018). β-Glucans: Multi-functional modulator of wound healing. Molecules.

[42] Frenkel J.S. (2014). The role of hyaluronan in wound healing. Int. Wound J..

[43] Litwiniuk M., Krejner A., Speyrer M.S., Gauto A.R., Grzela T. (2016). Hyaluronic Acid in Inflammation and Tissue Regeneration. Wounds.

[44] Chen W.Y., Abatangelo G. (1999). Functions of hyaluronan in wound repair. Wound Repair Regen..

[45] Della Sala F., Longobardo G., Fabozzi A., di Gennaro M., Borzacchiello A. (2022). Hyaluronic Acid-Based Wound Dressing with Antimicrobial Properties for Wound Healing Application. Appl. Sci..

[46] Lin Z.Q., Kondo T., Ishida Y., Takayasu T., Mukaida N. (2003). Essential involvement of IL-6 in the skin wound-healing process as evidenced by delayed wound healing in IL-6-deficient mice. J. Leukoc. Biol..

[47] Choudhary V., Choudhary M., Bollag W.B. (2024). Exploring skin wound healing models and the impact of natural lipids on the healing process. Int. J. Mol. Sci..

[48] Gajbhiye S., Wairkar S. (2022). Collagen fabricated delivery systems for wound healing: A new roadmap. Biomater. Adv..

[49] Pastar I., Stojadinovic O., Yin N.C., Ramirez H., Nusbaum A.G., Sawaya A., Patel S.B., Khalid L., Isseroff R.R., Tomic-Canic M. (2014). Epithelialization in wound healing. Adv. Wound Care.

[50] Raffetto J.D. (2016). Pathophysiology of wound healing and alterations in venous leg ulcers-review. Phlebology.

[51] Ray P., Singh S., Gupta S. (2019). Topical antimicrobial therapy: Current status and challenges. Indian J. Med. Microbiol..

[52] Breijyeh Z., Karaman R. (2024). Antibacterial activity of medicinal plants and their role in wound healing. Futur. J. Pharm. Sci..

[53] Liu E., Gao H., Zhao Y., Pang Y., Yao Y., Yang Z., Zhang X., Wang Y., Yang S., Ma X. (2022). The Potential Application of Natural Products in Cutaneous Wound Healing: A Review of Preclinical Evidence. Front. Pharmacol..

[54] Cheng Y.C., Li T.S., Su H.L., Lee P.C., Wang H.D. (2020). Transdermal delivery systems of natural products applied to skin therapy and care. Molecules.

[55] Lagoa T., Queiroga M.C., Martins L. (2024). An overview of wound dressing materials. Pharmaceuticals.

[56] Criollo-Mendoza M.S., Contreras-Angulo L.A., Leyva-López N., Gutiérrez-Grijalva E.P., Jiménez-Ortega L.A., Heredia J.B. (2023). Wound Healing Properties of Natural Products: Mechanisms of Action. Molecules.

[57] Abreu H., Zavadinack M., Smiderle F.R., Cipriani T.R., Cordeiro L.M.C., Iacomini M. (2021). Polysaccharides from *Pleurotus eryngii*: Selective extraction methodologies and their modulatory effects on THP-1 macrophages. Carbohydr. Polym..

[58] Đorđevski N., Abdullahi I.U., Zengin G., Božunović J., Gašić U., Ristanović E., Ćirić A., Nikolić B., Stojković D. (2023). Chemical and biological investigations of *Allium scorodoprasum* L. flower extracts. Pharmaceuticals.

[59] Petrović J., Kovalenko V., Svirid A., Stojković D., Ivanov M., Kostić M. (2021). Individual stereoisomers of verbenol and verbenone express bioactive features. J. Mol. Struct..

[60] Petrović J., Glamočlija J., Milinčić D.D., Doroški A., Lević S., Stanojević S.P., Kostić A.Ž., Minić D.A.P., Vidović B.B., Plećić A. (2024). Comparative Chemical Analysis and Bioactive Properties of Aqueous and Glucan-Rich Extracts of Three Widely Appreciated Mushrooms: *Agaricus bisporus* (JE Lange) Imbach, *Laetiporus sulphureus* (Bull.) Murill and *Agrocybe aegerita* (V. Brig.) Vizzini. Pharmaceuticals.

